# The impact of chromophore choice on the assembly kinetics and primary photochemistry of a red/green cyanobacteriochrome

**DOI:** 10.1039/d1cp02696h

**Published:** 2021-08-04

**Authors:** David Buhrke

**Affiliations:** Department of Chemistry, University of Zürich Zürich Switzerland david.buhrke@chem.uzh.ch

## Abstract

Cyanobacteriochromes (CBCRs) are bi-stable photoreceptor proteins with high potential for biotechnological applications. Most of these proteins utilize phycocyanobilin (PCB) as a light-sensing co-factor, which is unique to cyanobacteria, but some variants also incorporate biliverdin (BV). The latter are of particular interest for biotechnology due to the natural abundance and red-shifted absorption of BV. Here, AmI-g2 was investigated, a CBCR capable of binding both PCB and BV. The assembly kinetics and primary photochemistry of AmI-g2 with both chromophores were studied *in vitro*. The assembly reaction with PCB is roughly 10× faster than BV, and the formation of a non-covalent intermediate was identified as the rate-limiting step in the case of BV. This step is fast for PCB, where the formation of the covalent thioether bond between AmI-g2 and PCB becomes rate-limiting. The photochemical quantum yields of the forward and backward reactions of AmI-g2 were estimated and discussed in the context of homologous CBCRs.

## Abstract 2

The assembly reaction and primary photochemistry of a cyanobacteriochrome photoreceptor with two different chromophores were investigated with transient absorption spectroscopy.

## Graphical abstract

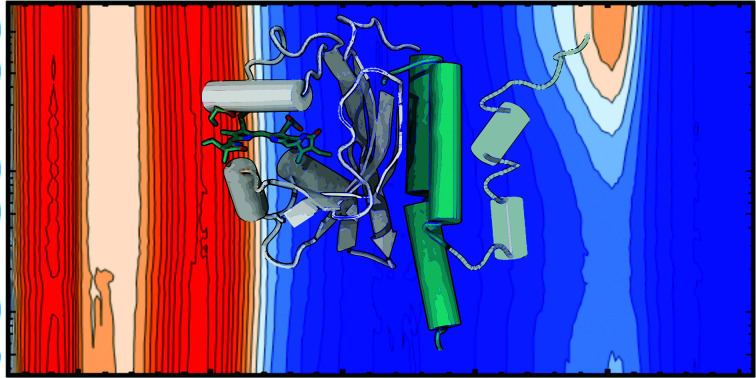

## I Introduction

Optogenetic techniques use external light sources to control and detect biological processes on a cellular level. This is achieved by introducing signaling photoreceptor proteins into target cells that are naturally not light sensitive.^1^ Bilin photoreceptors, among others, are especially useful for controlling relatively slow cellular processes such as protein expression or post-translational modification.^2,3^ One sub-class of bilin-photoreceptors, the cyanobacteriochromes (CBCRs), are promising building-blocks for such applications, because they have diverse absorption properties spanning from the ultraviolet to the near infrared, and can be coupled with a variety of output modules.^4^ Some recent examples are CBCR-based cyclases,^5^ histidine kinases^6^ or fluorescent markers.^7^ CBCRs can be large multidomain proteins that often include multiple photosensory GAF (cGMP-specific phosphodiesterases/adenylyl cyclases/FhlA) domains linked together in a chain, each hosting a bilin chromophore.

**Fig. 1:**
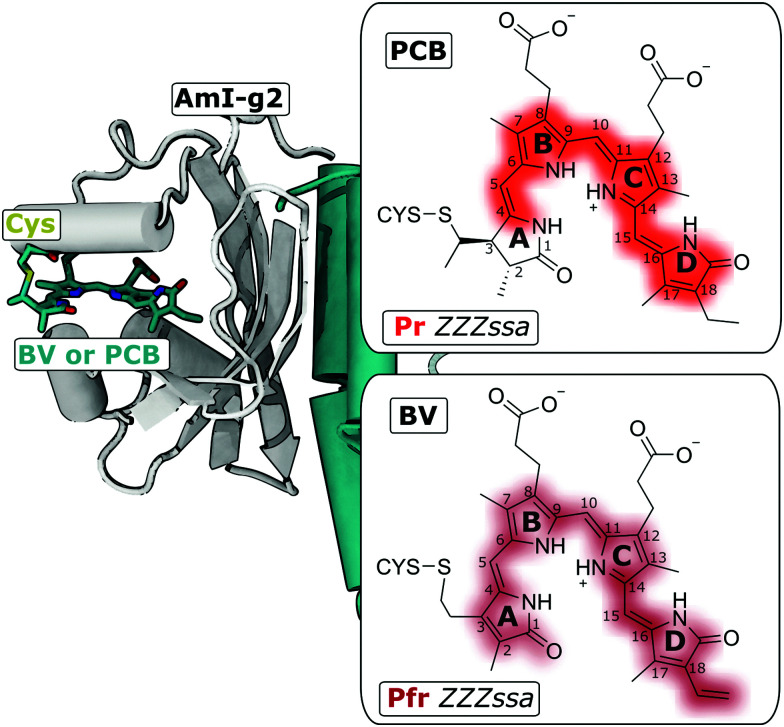
Am1-g2 binds BV or PCB to a conserved Cys residue. Left side: Crystal structure of the homologous GAF domain from the CBCR AnPixJ (with PCB, PDB entry 3W2Z). Right: The Lewis structures of the PCB and BV chromophores attached to the Cys residue in the *ZZZssa* configuration (Pr and Pfr states, respectively).

Here, the isolated GAF domain AmI-g2 (Fig. 1) was investigated, which can be used for light-regulation of gene expression, *e.g.* in yeast.^8^ AmI-g2 (abbreviated; in the original works called Am1-c0023g2) was discovered in the chlorophyll *d*-bearing cyanobacterium *Acaryochloris marina*^9,10^ and is a member of the red/green lineage of CBCRs. These proteins absorb red light and photoconvert reversibly to a green-absorbing signaling state. Among other red/green CBCRs, AmI-g2 is of special interest for biotechnological applications, because it can not only incorporate phycocyanobilin (PCB), a bilin that only cyanobacteria can synthesize, but also biliverdin (BV), which is abundant in many other potential host cells (*e.g.* mammalian tissues). Either of these chromophores form a covalent thioether linkage with a conserved (canonical) cysteine side chain,^11,12^ and recent studies suggested that a different carbon atom is bound in BV or PCB, respectively (Fig. 1, right insets).^13^ BV includes two more unsaturated C=C bonds, resulting in an extended π-electron conjugation compared to PCB. Therefore the visible absorption spectrum of the BV-bound protein is further red-shifted, which allows for photoactivation in deeper layers of biological tissue.^14^ Due to this shift, the dark adapted state is called Pr (red-absorbing parent state) in the PCB-bound protein, and Pfr (far-red) in the BV variant. Please note that the term “Pfr” for BV-binding CBCRs only refers to the position of the absorption maximum and not the stereochemistry of the chromophore.^8,9^ These terms are defined differently in the context of phytochromes, where “Pfr” is usually associated with various chromophores, including both PCB and BV, adopting a specific (*ZZEssa*) stereochemistry.^15,16^

The natural abundance and red-shift are the main reasons why BV-binding variants are preferred for optogenetic applications. However, when the PCB- and BV-bound variants of AmI-g2 were screened for their dynamic range in regulating the expression of a reporter protein in yeast, the BV-binding protein performed much worse than the PCB variant.^8^ There are many conceivable microscopic reasons for these findings, and two of them were investigated here. First, the efficiency of chromophore uptake can be different, which effectively limits the production of holo-protein in the cells, and second, different photochemical properties of the holo-proteins could alter the formation of the functional active state.

## II Materials and Methods

### Protein expression and purification

The apo AmI-g2 expressing cells were generated by transforming *E. coli* BL21 with a pET-30a(+) vector (kanamycin resistance) containing the sequence for apo-AmI-g2 in the open reading frame (GenScript Biotech, Piscataway Township NJ, USA). The apo- and holo-proteins were expressed and purified as described previously.^17^

### *In vitro* assembly experiments

2 mL of 3 μM bilin in aqueous buffer were prepared in a 1 cm pathlength quartz cuvette and continuously stirred throughout the experiment. The buffer was based on standard PBS with additional 5 mM EDTA to scavenge metal ions and 5 mM β-mercaptoethanol as reducing agent to keep the canonical Cys thiol in a reduced state. The initial concentrations of the bilins were checked by monitoring the absorption at the Q-band maximum (PCB: 605 nm, BV: 690 nm). The respective extinction coefficients at these wavelengths *ε*(BV) = 30.8 mM^−1^ cm^−1^ and *ε*(PCB) = 37.9 mM^−1^ cm^−1^ were taken from the literature.^18^ Then, 500 μL of apo-AmI-g2 stock solution (23 μM) in the same buffer were added to obtain 1.9× molar excess of protein in the reaction mixture (4.6 μM protein, 2.40 μM bilin). The initial dead time of the experiment was dictated by the mixing period, and could be determined by monitoring the intensity rise of the protein marker band at 280 nm (*ca.* 1 s). Mixing and the following reaction were monitored with an OCUSB4000 fiber spectrometer (Ocean Insight, Stuttgart, Germany) equipped with a matching halogen/deuterium dual light source (DH-2000-BAL). The experiments were performed in the dark to prevent any unwanted photochemistry, and the incident measurement light was minimized by using a shutter which only opened during the integration period of 22 ms for each recorded spectrum. The integration time of the spectrometer and shutter in front of the measurement light were both synchronized to a pulse-delay generator. For PCB, spectra were recorded every 200 ms, for BV, every 2 s. To reduce the size of the large data sets and improve the S/N ratio, 7 pixels were binned along the wavelength axis which resulted in a spectral resolution of *ca.* 1 nm, and logarithmic binning was applied along the time axis.

### Pump–probe spectroscopy

The samples were kept in the Pr or Pfr states by illumination with a green LED array (LIU525B, Thorlabs, Newton, MA, USA) or by keeping them in darkness. Samples were illuminated with red and far-red laser diodes to prepare the Pg state in the case of PCB-adduct (HL6750MG, Thorlabs), and the Po state in case of the BV-adduct (HL7302MG) in a sample reservoir. Electronically synchronised fs-lasers were used for the generation of the pump and probe pulses^19^ as described earlier^20^ with only minor adaptations in the pump light for the present study. Here, compressed pulses with 8 ps duration and with a power of 0.5 μJ at 385 nm were used for excitation of all samples. The spot sizes were *ca.* 120 (pump) and 90 μm (probe) and the beams were polarized in magic angle relative to each other. The time resolution of 10 ps was dictated by the jitter of the electronic synchronisation.^19^

## III Results

### Assembly with PCB

**Fig. 2:**
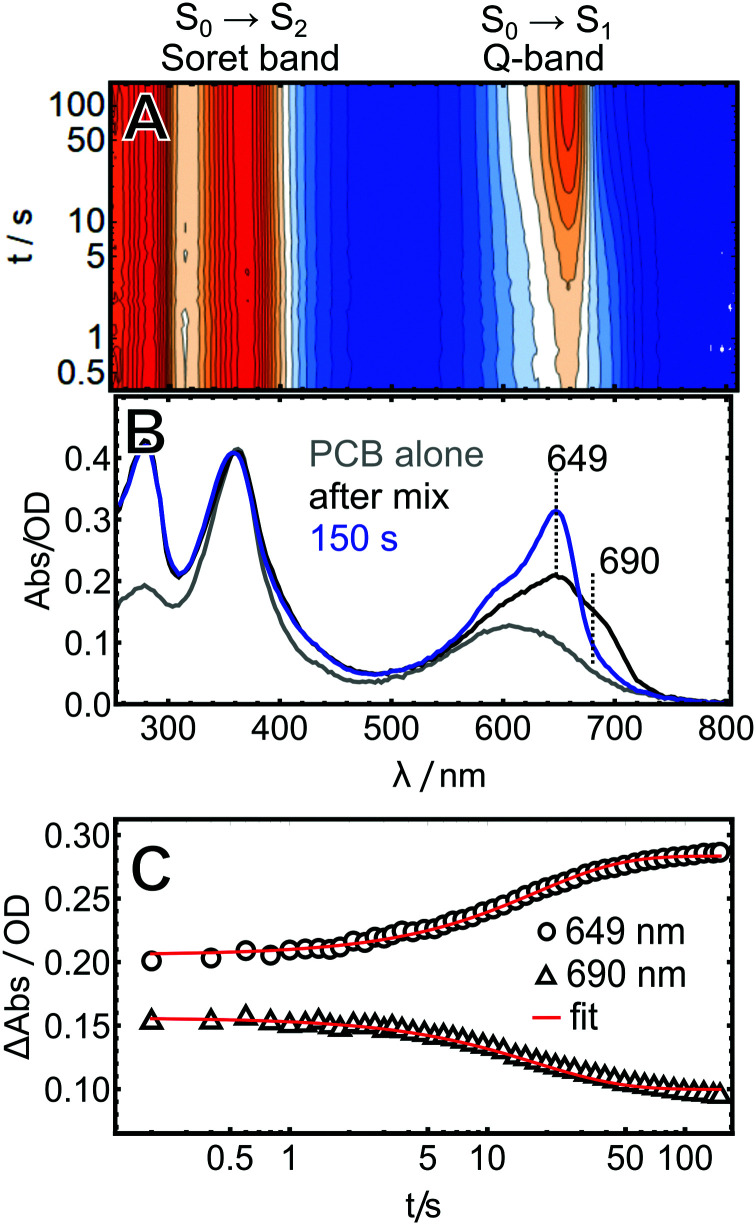
*In vitro* assembly of AmI-g2 with PCB. (A) Heatmap of the time-resolved data, (B) absorbance spectra of PCB in buffer (grey trace) and the AmI-g2/PCB reaction mixture at selected time points (blue and black traces), and (C) kinetic traces at the selected wavelengths.

The assembly reactions of AmI-g2 with PCB and BV were investigated *in vitro* with an inexpensive home-built setup including a fiber-spectrometer (see Materials and Methods for details). After mixing, spectra were recorded at fixed delay times until no further changes were noticed, which was after *ca.* 150 s in the case of PCB. The data are visualized in a heatmap plot in Fig. 2A.

Experimental and theoretical studies showed that free PCB in solution is very flexible and can adopt a variety of different conformations by twisting around its three methine bridges.^21,22^ The by-far dominant species in this ensemble are left- and right-handed helical structures denoted as *ZZZsss*. Here, all methine bridge double bonds (C4=C5, C10=C11 and C15=C16) are in *cis*(*Z*) and the single bonds (C5–C6, C9–C10 and C14–C15) in *syn*(*s*) configuration. Inside the protein, PCB is bound in a *ZZZssa* configuration with the C14–C15 bond in an *anti* position (Fig. 1). Helical PBC exhibits a characteristic strong Soret-band around 385 nm and a distinctly weaker Q-band with a broad, unstructured maximum around 600 nm (Fig. 2B).^18,23^ The spectrum changes drastically over the course of the experiment, with a strong increase in the Q-band extinction at 649 nm, which is characteristic for many PCB-binding CBCRs and phytochromes in the Pr state. This peak is already detected in the first spectrum taken 0.2 s after mixing, indicating that already here a fraction of the final holo-protein is formed. Additionally, an intermediate shoulder is observed in the red flange of the Q-band at *ca.* 690 nm. This feature disappears over the course of the experiment, while the main band at 649 nm continues to grow in, following the same kinetics. A global fit of the data with a single exponential function afforded a time constant of 18 s for this process.

### Assembly with BV

**Fig. 3:**
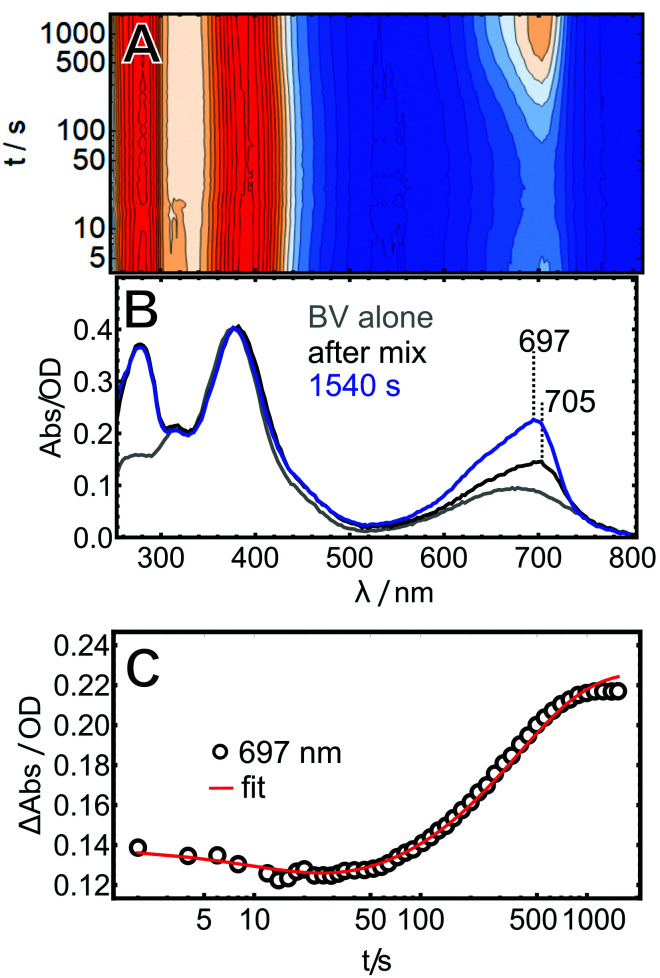
*In vitro* assembly of AmI-g2 with BV. (A) Heatmap of the time-resolved data, (B) absorbance spectra of BV in buffer (grey trace) and the AmI-g2/BV reaction mixture at selected time points (blue, black, and green traces), and (C) kinetic trace at the Q-band maximum of holo-AmI-g2 (BV).

Similar to PCB, BV adopts many different, but predominantly helical conformations in solution,^24,25^ and is typically found in the *ZZZssa* configuration in CBCRs or phytochromes after assembly in the dark. However, when the same *in vitro* assembly experiment was performed with BV instead of PBC, some pronounced differences were noted (Fig. 3).

**Fig. 4:**
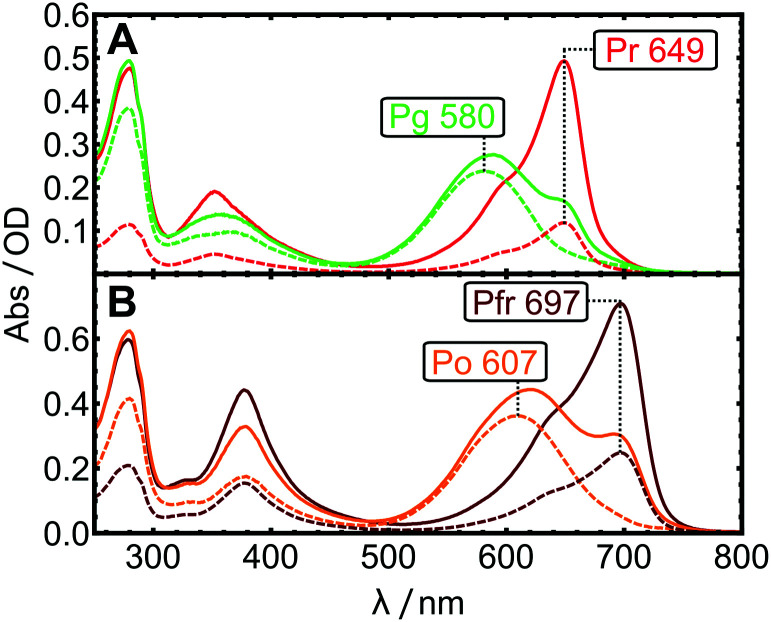
Steady-state absorption spectra. (A) AmI-g2 PCB in the Pr (red solid line) and in photostationary equilibrium (green solid line). (B) AmI-g2 BV in the Pfr (dark red solid line) and photostationary equilibrium (orange solid line). The individual contributions of pure spectra to the equilibria were calculated as described in the text (dashed lines).

First, all absorption bands are red-shifted due to the extended π-electron system. This applies for the free bilin in solution and the holo-proteins (see Fig. 1 for Lewis structures and Fig. 4 for spectra of purified holo-proteins).

Second, the entire assembly processes is slower by roughly a factor of 10× and only finished after *ca.* 1500 s (note the different time axes in Fig. 2 and 3).

Third, the time-resolved data reveal at least two processes that have opposite effect on the intensity in the Q-band region. After an instantaneous increase upon mixing, the intensity around 700 nm decreases over the course of *ca.* 10 s and increases again during the rest of the experiment.

Fourth, the Q-band maxima of the early spectra (up to *ca.* 30 s) exhibit a slightly red-shifted maximum at 705 nm, which shifts down to 697 nm at later times.

The BV data were globally fitted with two exponential terms, affording time constants of 14 and 385 s.

### Photostationary equilibrium

The spectra of the purified holo-proteins are shown as red- and dark-red solid lines in Fig. 4.

Upon illumination with red light, both AmI-g2 variants photoswitch and form long-lived photoproduct states with blue-shifted Q-band maxima. These states are consequently called Pg (green-absorbing) and Po (orange), for PCB and BV, respectively. While Pr and Pfr are easily obtained by storing the sample in the dark or under green light, it is not straightforward to prepare samples in the photoproduct state as starting point for time-resolved experiments. The long-lived photoproduct can be enriched by background illumination, but this establishes photostationary equilibria that include significant residual Pr/Pfr contributions (solid orange and green lines). In some CBCRs like Slr-g3, the fraction of residual Pr can be made very small (<5%) by exciting the sample in a region where photoproduct absorption is minimal.^26^ In the case of AmI-g2, the significant overlap between the absorption spectra of the dark-adapted (*S*_dark_) and photoproduct states (*S*_phot_) leads to significant contributions of *S*_dark_ to the equilibrium spectra *S*_equil_ for both chromophores. Different light-sources were screened to minimize the amount of residual Pr and Pfr, and the best results are shown in Fig. 4, obtained with laser diodes emitting at 685 nm (PCB) and 730 nm (BV). By subtracting according to *S*_phot_ = *S*_equil_ − *s* × *S*_dark_, the pure spectrum *S*_phot_ can be approximated. The subtraction factor *s* was determined iteratively in order to: (1) completely remove the contribution of *S*_dark_ evaluated at the prominent marker band at 649 or 697 nm, and (2), avoid over-subtraction indicated by negative or sharp features. Subtraction factors of 0.35 and 0.22 yielded the pure Po and Pg spectra (dotted lines in Fig. 4).

### Primary photochemistry

**Fig. 5:**
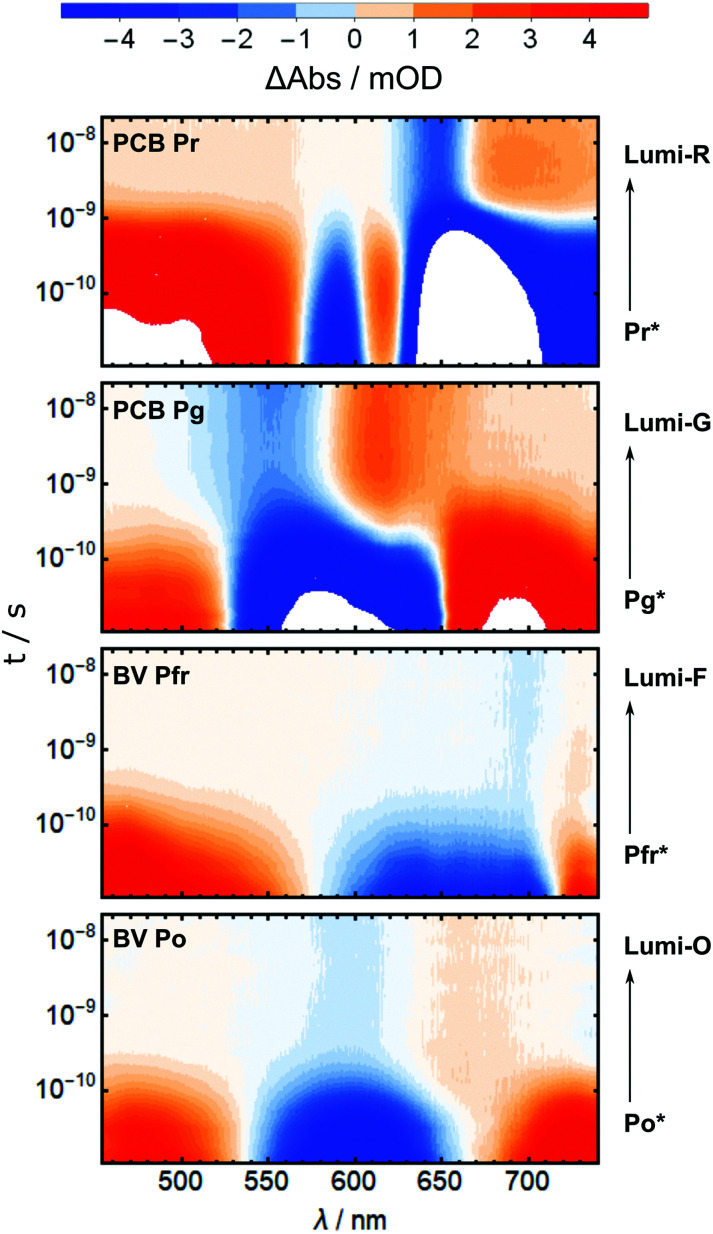
Heatmaps of the pump–probe data for the Pr, Pg, Pfr and Po states (top to bottom).

The primary photochemistry of AmI-g2 in the Pr, Pg, Pfr and Po states was investigated with transient absorption spectroscopy in pump–probe experiments. As discussed beforehand, the residual contributions of Pr or Pfr need to be subtracted from the Pg and Po data. In the case of the pump–probe data, *s* was adjusted by multiplication with the ratio of extinction coefficients at the pump wavelength (385 nm). Subtraction of the entire TA datasets was only successful when they were recorded in immediate succession and the individual measurement time was kept short (*ca.* 20 min per dataset). Even smallest adjustments between the measurements, *e.g.* in the overlap of pump and probe beams, disturbed the sensitive calibration of the dispersive experiments such that subtraction afforded spectral artifacts. A time-window from 10 ps to 20 ns was chosen for all pump–probe experiments, which fully covers the decay of the electronic excited states (ES, denoted by an asterisk *e.g.* Pr*) to the respective primary ground state intermediate called Lumi (*e.g.* Lumi-R in the case of Pr* decay). Fig. 5 shows the results in a heatmap representation and reveals that there is indeed one dominating process in this time window for all four experiments.

**Fig. 6:**
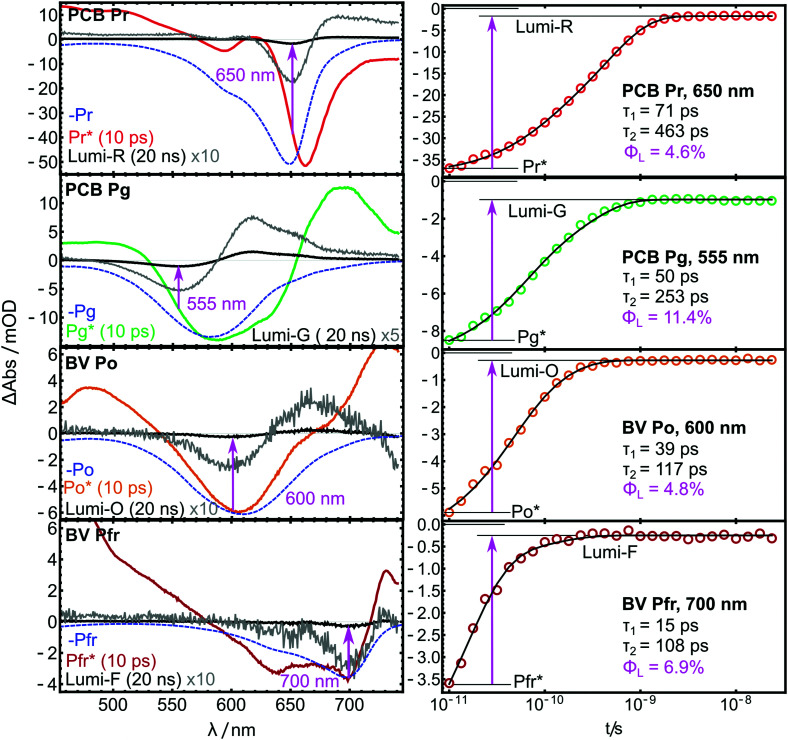
TA spectra (left) and kinetic traces (right) of the AmI-g2 PCB and BV adducts from the pump–probe experiments.

In all TA data, the early 10 ps spectra display more than 10× larger amplitudes than the Lumi spectra around 20 ns. Note that in Fig. 6, the Lumi spectra (black lines) were scaled up by factors of 5×–10× (grey lines) for better visibility. This difference in amplitudes is observed in most bilin photoreceptors and is mainly attributed to the low photochemical quantum yield for the isomerisation reaction (*Φ*_L_), *i.e.* most of the excited molecules return to the ground state without isomerisation. All pump–probe difference spectra contain overlapping contributions from ground state bleach (GSB, negative) and product absorption, either from the excited state (ESA, positive), or the Lumi photoproduct (PA, positive). Additionally, stimulated emission (negative) can occur from the ES.

The spectra reported here are highly similar to previously published results of many related PCB- and BV-binding CBCRs. Their spectral shapes and often complex kinetics have been analyzed in much detail elsewhere and indicate a complex interplay of different processes and ES decay channels.^26–32^ Global fits with two exponential terms and a non-decaying component were sufficient to account for these complex kinetics in the investigated time window. The resulting time constants *τ* are given as insets in Fig. 6. Pronounced differences are observed between the PCB- and the BV-binding variants: Pfr* decays more than 4× faster than Pr*, and Po* approximately 2.5× faster than Pg*.

### Estimation of quantum yields

If the bleach and absorption bands were perfectly separated, *Φ*_L_ could simply be calculated as the fractions of GSB still present in the Lumi spectra. However, the overlap with the positive product absorption signals partially cancels the bleach bands in both ES and Lumi states to a different extent. In the ES, additional interference from stimulated emission is expected. Therefore, the values for *Φ*_L_ can only be approximated by calculating the GSB ratio (magenta insets in Fig. 6), but the shape of the spectra allow to evaluate the quality of the approximation. In the Lumi spectra, there is no stimulated emission, and so the overlap with PA can only lead to underestimation of the bleach. Here, a strong overlap will further shift the minimum of the GSB. In Fig. 6, this effect is highlighted by plotting the inverted steady-state absorption spectra (blue dashed lines) together with the pump–probe data. A significant GSB shift is only visible in the Lumi-G and Pr*. Here, the strong absorption of Lumi-G at 580 nm produces a downshift of the GSB to 555 nm, while in the other Lumi data, the overlap of PA and GSB does not lead to such pronounced shifts. Thus, *Φ*_Lumi-G_ is probably quite underestimated by this approach, while the error in the other data appears to be smaller (although still present). A similar logic applies to the Pr* spectrum, where both ESA and stimulated emission distort the GSB with different signs, and partially cancel each other. Generally, the GSB bands are less distorted in the BV-bound variant, and the approximations hold better compared to PCB.

*Φ*_L_ further depends on the excitation wavelength, but in case of the closely related red/green CBCR Slr-g3, similar GSB ratios were observed independent of Q-band^26^ or Soret-band excitation,^20^ which is an indication that this factor plays only a minor role in red/green CBCRs. The values for *Φ*_L_ were calculated from the fit at the Lumi GSB minima.

## IV Discussion

### Assembly mechanism

**Fig. 7:**
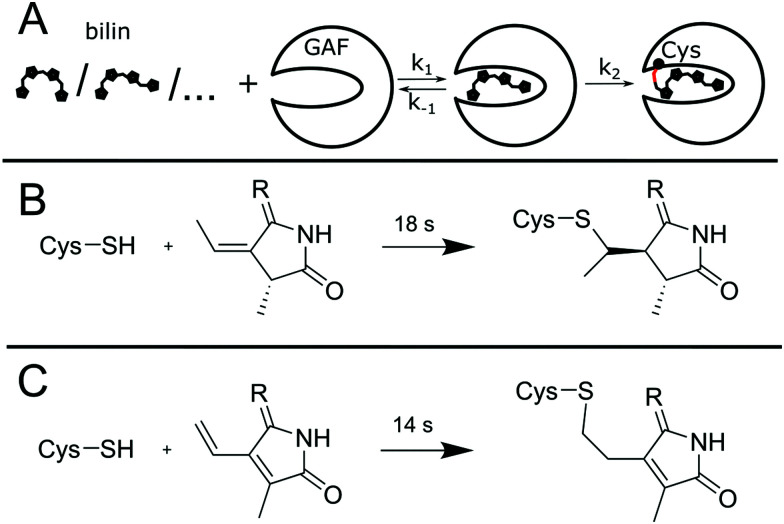
Assembly reaction of AmI-g2 with bilin chromophores. (A) Two-step reaction scheme. Lower panels: Comparison of the attachment reactions with PCB (B) and BV (C).

The assembly of bilin holo-proteins is a complex reaction, but a simple two-step model can be used to interpret the spectroscopic data and understand the reaction mechanism (Fig. 7A). In this model, the bilin must enter the binding pocket first with *k*_1_, and only then the spatial proximity allows for the formation of the covalent thioether bridge with *k*_2_. Here, the Cys thiol adds either to an exocyclic double bond of PCB (Fig. 7B) or the vinyl group of BV (Fig. 7C), in a process that is facilitated by the surrounding amino acids. The thiol addition shortens the conjugated system by one double bond and therefore leads to spectral blue-shifts. Here, the visible absorption spectrum acts as a sensitive marker for the Cys-addition reaction as recently also demonstrated for the photo-labile thioether bond in the blue/green CBCR TePixJ.^33^

The assembly curve with PCB qualitatively resembles results from a comparable experiment with the cyanobacterial phytochrome Cph1, where a similar red-shifted intermediate was observed directly after mixing protein and PCB. In this study, Zn^2+^ staining was used to correlate the disappearance of the red shoulder with the progress of covalent attachment.^23^ In another related biliprotein, small amounts of PCB could still be incorporated after substitution of the canonical cys, and a characteristic absorption band around 690 nm was assigned to the non-covalent species.^34^ Unfortunately, a similar experiment with the red/green CBCR AnPixJ-g2 showed no non-covalent binding when the Cys residue was substituted by Ala.^11^ In this context, our results imply that PCB uptake and formation of the non-covalent intermediate (*k*_1_) is largely finished within the dead time. The 18 s process corresponds to the thioether formation, which is an irreversible reaction that can be approximated reasonably well by the first-order kinetics and the exponential fit.

**Fig. 8:**
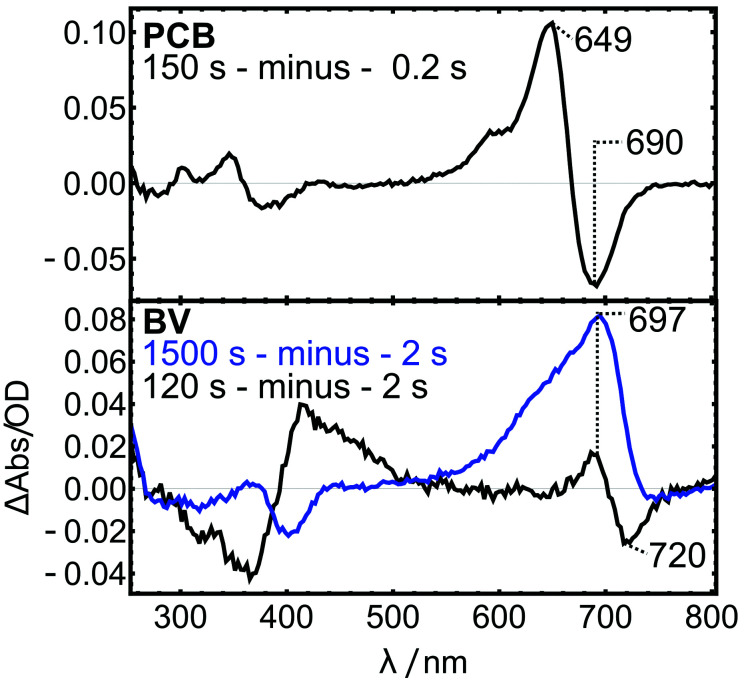
Difference spectra at selected time points for the *in vitro* assembly experiments.

In the case of BV, the transient blue-shift is less pronounced. For a better visualization, difference spectra at two selected time-points are compared to PCB (Fig. 8). After 120 s, the Q-band region shows a clear difference signal at 720/697 nm, qualitatively similar to the thioether formation in the PCB variant, indicating that such a reaction already takes place in the first phase of the experiment. The difference spectrum after 1500 s is dominated by the positive signal of the covalently bound protein, which compensates the small negative bleach band of the non-covalent intermediate. These results indicate that the concentration of the blue-shifted intermediate is low at all times, which is typically the case when the second step (thioether formation) is faster than rate-limiting first step (the uptake of BV by the protein). This interpretation is in line with the finding that the exponential fit deviates from the data at later times, because the rate-limiting BV uptake is a second order process and therefore does not follow exponential kinetics.

The significantly smaller red-shift (*ca.* 8 nm compared to 40 nm in PCB) of the noncovalent intermediate was also observed in two earlier studies of different bacteriophytochromes with different Cys substitutions.^35,36^ In these studies, binding of BV *via* addition to the terminal C-atom of the vinyl group also afforded only minor absorption shifts compared to the noncovalent form, and the introduction of a second Cys residue led to a second thioether formation, saturation of Ring A, and larger spectral blue-shifts in line with the present findings for PCB.

### Photochemical quantum yields

**Fig. 9:**
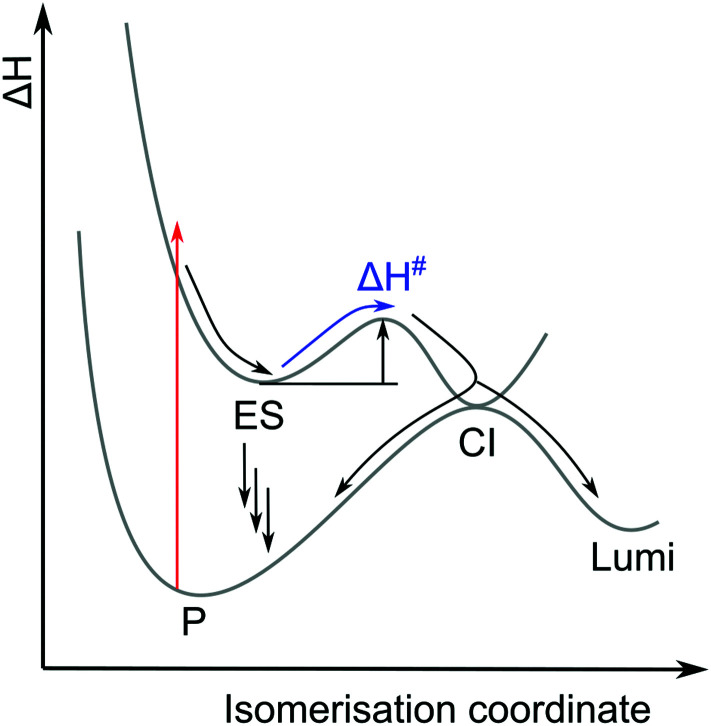
General energy-diagram for photochemical isomerisation involving a barrier on the ES.

The ES decay kinetics of all four reactions extend into hundreds of picoseconds, which indicates significant energy barriers compared to the sub-picosecond photoisomerisation of other molecules such as rhodopsin or azobenzenes.^37,38^ The rate-determining step is the crossing of that barrier on the ES, which is related to an activation energy needed to reach a transition state #. This finding is common for bilin-photoreceptors and often visualized in energy-diagrams like in Fig. 9.^27,30^

In the diagram it is visualized by arrows that *Φ*_L_ depends on the rates of all competing ES decay pathways, such as the direct decay of ES to P by internal conversion, fluorescence or stimulated emission, and also the branching ratio at the conical intersection (CI) after barrier-crossing. Fluorescence plays a role especially in the Pr with a quantum yield of 3%, while the Pfr emits only weakly (0.2%), and no values could be determined for Pg and Po.^10^ While the complex decay kinetics that are often measured for CBCRs can be fitted to target models based on such diagrams, it is not possible to invert this underdetermined problem and determine *Φ*_L_ directly from the decay kinetics.

Albeit the values that we obtain by comparing the GSB bands include a certain error, a comparison with published data shows that values for *Φ*_L_ can differ by orders of magnitude between different red/green CBCRs. Some, like AnPixJ-g2 or NpR6012g4, have large *Φ*_L_ for both Lumi-R (*ca.* 40%) and Lumi-G (*ca.* 55%).^27,31,39^ AmI-g2 ranges on the lower end of the spectrum for the *Φ*_L_ in both directions, with values <10% and amplitudes comparable *e.g.* to Lumi-R formation in Slr1393-g3.^20,26,40^ In the overall context of different red/green CBCRs, AmI-g2 has very low quantum yields both directions, which are comparable in the PCB and BV variants. These values do not correlate directly with the ES lifetimes due to the high complexity of the ES decay reactions and many different decay channels.

## V Conclusions

The assembly reaction with PCB is roughly 10× faster than with BV. This finding is important for biotechnological application of AmI-g2 in cellular environments, where porphyrins and other small hydrophobic molecules compete with bilins for binding to the apo-proteins.^35,41^ Therefore, the rate of the bilin uptake (*k*_1_) is an important factor playing into the effective concentration of holo-protein *in vivo*,^42^ and is probably at least one of the reasons why the PCB-binding variant performs better as an optogenetic tool in yeast.^8^ We also showed that AmI-g2 has very low photochemical quantum yields in both photoswitching directions compared to other red/green CBCRs, independent of which bilin was used. These values are also important for applications in cells, because their ratio, together with the spectral overlap, determines how many activated molecules will be present under photostationary conditions. In the case of AmI-g2, both of these factors result in relatively large residual contributions of Pr (*ca.* 22%) and Pfr (*ca.* 35%) to the steady state spectra.^8^ However, the connection between the residual contributions and the activity as optogenetic tool is not straightforward, as the low quantum yields for the deactivation also lead to an increased light-stability of active state, which could even turn out to be beneficial under constant-illumination conditions.

## Data availability

The data are openly available on Zenodo DOI: 10.5281/zenodo.5163608.

## Conflicts of interest

There are no conflicts of interest to declare.

## Supplementary Material
